# Reduced RhoA expression enhances breast cancer metastasis with a concomitant increase in CCR5 and CXCR4 chemokines signaling

**DOI:** 10.1038/s41598-019-52746-w

**Published:** 2019-11-08

**Authors:** Gardiyawasam Kalpana, Christopher Figy, Miranda Yeung, Kam C. Yeung

**Affiliations:** Department of Cancer Biology, College of Medicine and Life Sciences, University of Toledo, Health Science Campus, Toledo, Ohio, 43614 USA

**Keywords:** Breast cancer, RHO signalling, Cancer microenvironment, Metastasis

## Abstract

The role of RhoA GTPases in breast cancer tumorigenesis and metastasis is unclear. Early studies within which mutations in RhoA were designed based on cancer-associated mutations in Ras supported an oncogene role for RhoA. However, recent whole-genome sequencing studies of cancers raised the possibility that RhoA may have a tumor suppression function. Here, using a syngeneic triple negative breast cancer murine model we investigated the physiological effects of reduced RhoA expression on breast cancer tumorigenesis and metastasis. RhoA knockdown had no effect on primary tumor formation and tumor proliferation, concurring with our *in vitro* findings where reduced RhoA had no effect on breast cancer cell proliferation and clonogenic growth. In contrast, primary tumors with RhoA knockdown efficiently invaded sentinel lymph nodes and significantly metastasized to lungs compared to control tumors. Mechanistically, the current study demonstrated that this is achieved by promoting a pro-tumor microenvironment, with increased cancer-associated fibroblasts and macrophage infiltration, and by modulating the CCL5-CCR5 and CXCL12-CXCR4 chemokine axes in the primary tumor. To our knowledge, this is the first such mechanistic study in breast cancer showing the ability of RhoA to suppress chemokine receptor expression in breast tumor cells. Our work suggests a physiological lung and lymph node metastasis suppressor role for RhoA GTPase in breast cancer.

## Introduction

Rho GTPases belong to the Ras GTPase superfamily and in humans consists of about 20 members. Among them, RhoA, Rac1 and CDC42 are the most studied Rho GTPases. These GTPases act as intermediate signal transducers for a diverse array of cell surface receptors such as cytokine receptors, cadherins, integrins, GPCR, and tyrosine kinases, and therefore regulate major cellular events such as cell polarity, cell migration, cell cycle progression, cytoskeleton rearrangements, vesicular trafficking, and gene expression. Aberrant Rho GTPase signaling results in neurological and immunological abnormalities, and malignant cell transformation^1^.

Like most other GTPases, RhoA and their activating proteins, guanine nucleotide exchange factors (GEFs), are commonly believed to be pro-proliferative and act as cancer oncogenes. The evidence for the oncogenic activity of RhoA was first reported using dominant negative and constitutively active RhoA mutants in fibroblasts. While activating RhoA mutants enhanced the transformation activity of a weakly oncogenic RAS mutant, a dominant negative RhoA mutant abrogated the oncogenic Ras-mediated transformation^2,3^. Later, RhoA was reported to be overexpressed in several epithelial human cancer tissue samples including, breast^4^, testicular^5^, liver, colorectal^6^, ovarian^7^ and gastric carcinoma^8^.

Recent advances in whole-genome sequencing have identified hitherto undiscovered mutations in cancers and identified recurrent loss-of-function and gain-of-dominant-negative function mutations in RhoA in lymphoma, leukemia and several solid tumors. These latest discoveries, therefore, suggested a possible tumor suppressor function for RhoA in cancers^9–12^. Indeed, studies with mouse models have demonstrated the tumorigenic and metastatic functions of the identified RhoA dominant-negative alleles in colorectal cancer^13^.

In addition, analysis of the RNA-seq data generated from The Cancer Genome Atlas (TCGA) project revealed high expression of several RhoGAP genes, which are the upstream negative regulators of RhoA, in basal-like breast cancer tumors raising the possibility that RhoGAPs are oncogenic^14^. The oncogenic role of a RhoGAP named ARHGAP18 in breast cancer was subsequently proven with mouse transplantation model. Importantly, ARHGAP18 was shown to enhance metastasis partly by inactivating RhoA^15^.

Although these recent advances in the field support a tumor suppressive role for RhoA, a comprehensive mechanistic analysis on altered RhoA expression in primary tumors and its subsequent effects on tumor microenvironment and metastasis is lacking. In this study, using the highly metastatic 4T1 orthotopic mouse model, which closely resembles the human triple negative breast cancer, we show that downregulation of RhoA expression increases breast cancer lung metastasis. Mechanistically our study suggests that by increasing the expression of chemokine receptors, CXCR4 and CCR5, decreased RhoA expression increases the proclivity of cancer cells for the sentinel lymph node and enables the cancer cells to have access to the circulation and metastasize.

## Results

### RhoA suppresses breast cancer cell invasion *in vitro*

**Figure 1:**
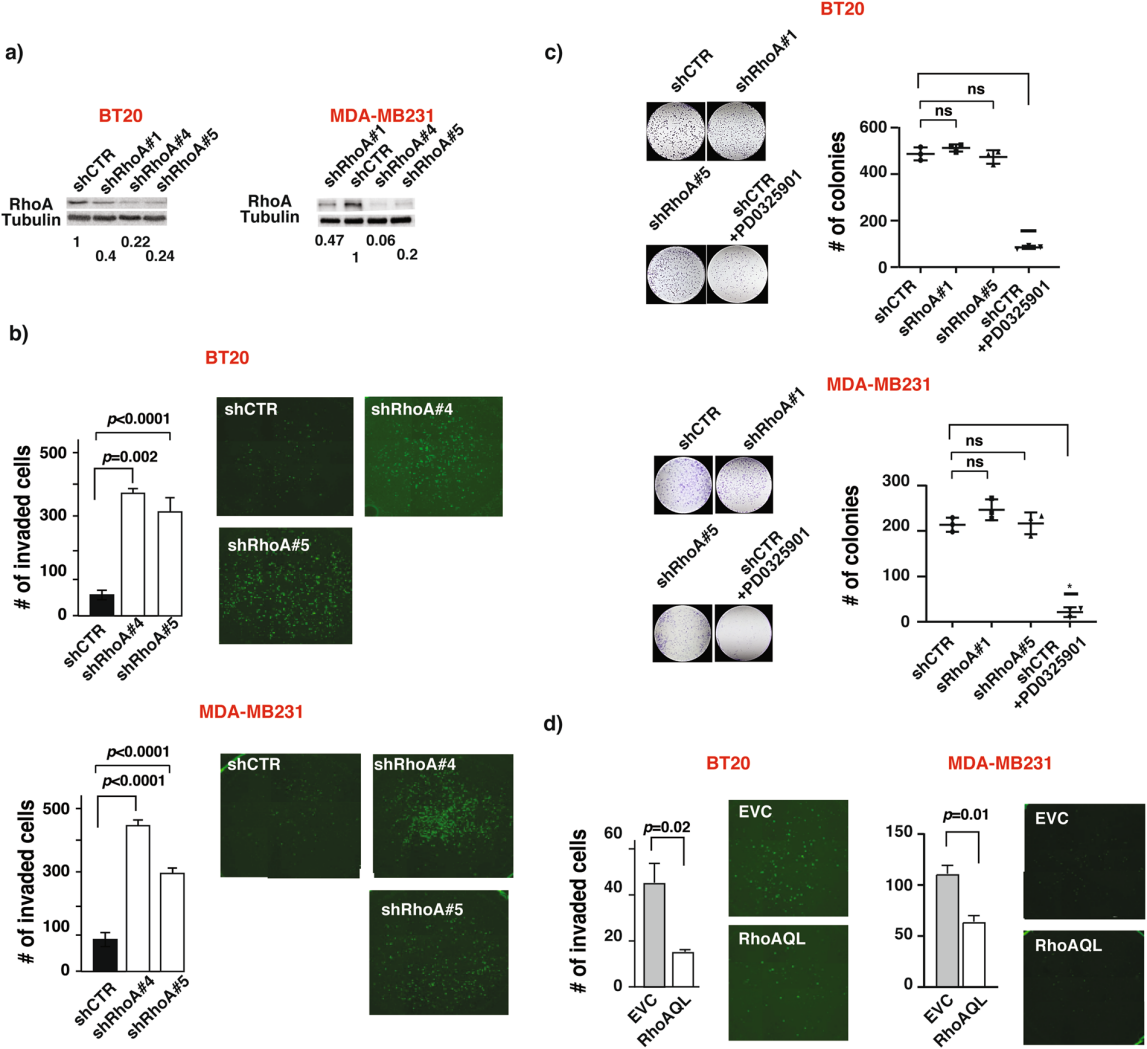
RhoA suppresses breast cancer cell invasion *in vitro*. (**a)** Representative western blots of the RhoA expression in BT20 (left) and MDA-MB231 (right) cell lines after lentiviral transduction of indicated constructs. Numbers are shown for quantified bands normalized with Tubulin. Complete blots are shown in Supplementary figures 4 and 5. (**b)** Number of invaded cells through Matrigel in indicated BT20 (top) and MDA-MB231 (bottom) cell lines (mean ± SE) and representative images of the stained invaded cells in indicated cell lines. Representative results of two independent assays. (**c)** Number of colonies for indicated BT20 (top) and MDA-MB231 (bottom) cell lines (mean ± SE) and representative images of the stained colonies in indicated cell lines. Representative results of two independent assays. PD0325901 used as positive control. (**d)** Number of invaded cells through Matrigel in indicated BT20 (left) and MDA-MB231 (right) cell lines (mean ± SE) and representative images of the stained invaded cells in indicated cell lines. *P <  0.05, ns- not significant, unpaired Student’s t-test (two-tailed).

Role of RhoA in breast cancer tumorigenesis and tumor progression have long been a topic of debate^16,17^. Although early work suggested a potential oncogenic role for RhoA, numerous recent studies start to challenge this notion^10,14,15,18^. To further investigate the role of RhoA in breast cancer, we stably down-regulated RhoA expression by lentiviral transduction of specific shRNAs (Fig. 1a) and measured the effects of the RhoA knockdown on cancer cells proliferation and invasion. Two different triple-negative breast cancer cell lines (TNBC) were used to ensure the observed effects were not cell type specific. While reduced RhoA expression significantly increased breast cancer cells invasion through Matrigel (Fig. 1b), it did not have a noticeable effect on cell proliferation as measured by two different assays (Fig. 1c and Supplementary Fig. 1). As a positive control for our proliferation assay, we treated breast cancer cells with a selective MEK inhibitor, PD0325901, and observed an expected near-complete suppression on colony formation in clonogenic growth assay (Fig. 1c).

We reasoned that if the effect of RhoA knockdown on breast cancer cell invasion is RhoA specific and physiologically relevant, we would expect to observe the opposite effect when RhoA expression or activity is increased. Indeed, stable expression of a constitutively active RhoA variant, RhoAQ63L, in both BT20 and MDA-MB231 considerably decreased their proclivity to invade (Fig. 1d). Together, these results suggested that in breast cancer cells, RhoA suppresses cell invasiveness.

### RhoA suppresses breast cancer lung metastasis burden in mice

**Figure 2:**
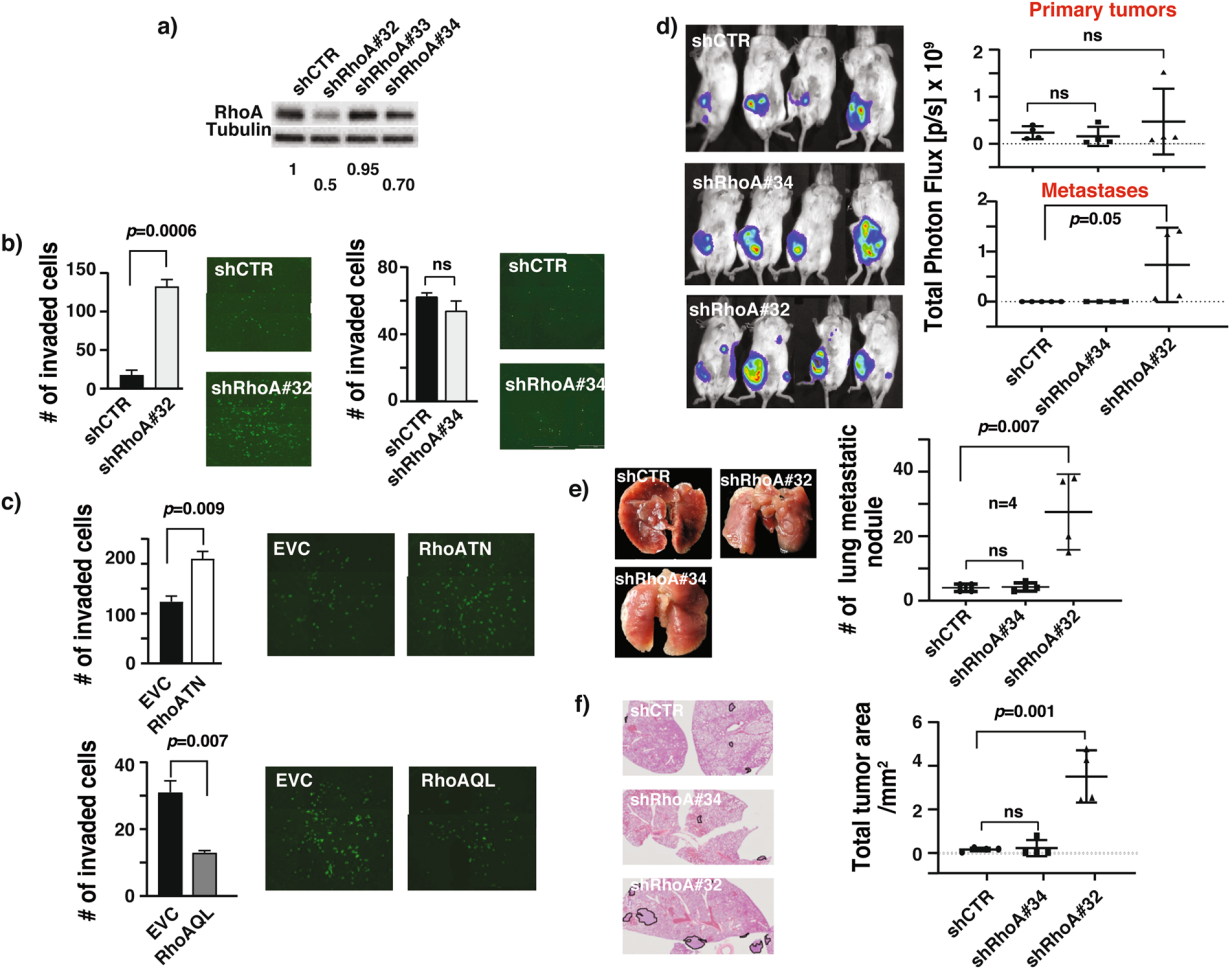
RhoA suppresses breast cancer lung metastasis burden in mice. (**a**) Representative western blots of the RhoA expression in 4T1 cell lines after lentiviral transduction of indicated constructs. Numbers are shown for quantified bands normalized with Tubulin. Complete blots are shown in Supplementary figures 6. (**b**) Number of invaded cells through Matrigel in 4T1 shRhoA 32 (left) and 4T1 shRhoA 34 (right) cell lines relative to their control cells lines (mean ± SE) and representative images of the stained invaded cells in indicated cell lines. Representative results of two or more independent assays. (**c**) Number of invaded cells through Matrigel in 4T1 RhoATN (top) and 4T1 RhoAQL (bottom) cell lines relative to their controls (mean ± SE) and representative images of the stained invaded cells in indicated cell lines. Representative results of two independent assays. (**d**) Representative BLI images of Balb/c mice orthotopically injected with 4T1 GFP-LUC cells carrying indicated lentiviral modifications, imaged 29 days after the implantation (left). Total photon flux quantification (mean ± SE) of BLI images shown on left for the signals in primary tumors (top) and metastases (bottom) (right). N = 4 for all groups. (**e**) Representative gross lung images of the mice orthotopically injected with 4T1 GFP-LUC cells carrying indicated lentiviral modifications, showing visible metastatic nodules (left). Number of lung metastatic nodules (mean ± SE) of these mice. N = 4. (**f**) Representative hematoxylin and eosin (H&E) staining of lung cross sections from above harvested lungs showing metastases highlighted with black (left). Total metastases tumor area per total lung area (mean ± SE) quantification of the H&E images (right). N = 4, ns- not significant *P <  0.05, unpaired Student’s t-test (two-tailed).

The effects of altered RhoA expression on breast cancer invasion *in vitro* raises the possibility that RhoA may have a causal role in suppressing breast cancer cells metastasis. Metastasis is a complex multimodal activity that involves both host and tumor cells and can only be adequately addressed with an animal model. To investigate the possible role of RhoA in breast cancer metastasis, we exploited the well-established 4T1 syngeneic murine breast cancer model^19,20^. The 4T1 TNBC cell line was originally derived from a spontaneous mammary tumor in a Balb/c mouse. Hence, it is widely utilized for syngeneic orthotopic mammary tumor allograft experiments. We generated a panel of lentiviral-mediated stable RhoA knockdowns in 4T1 cells with different levels of knockdowns, and used two of them, with a 30% (shRhoA 34) and a 50% (shRhoA 32) knockdown, for subsequent experiments (Fig. 2a). Concurring with human breast cancer cells results, reduced RhoA expression significantly increased 4T1 cells invasion through Matrigel in a dose-dependent manner (Fig. 2b). Significantly, similar effects were observed with stable expression of a dominant negative RhoAT19N, but the opposite effect was observed when the constitutively active RhoAQ63L was expressed in 4T1 cells (Fig. 2c). Collectively, these findings strongly suggested that the mouse 4T1 breast cancer cells could be used to investigate the role of RhoA in metastasis.

To address this question, we first engineered 4T1 cells to express a GFP-LUC hybrid protein to allow the growth and metastasis of the tumor to be monitored by luciferase bioluminescent imaging (BLI). Upon orthotopic implantation of 4T1 cells, all mice in RhoA knockdown and control groups developed primary tumors. In accord with our *in vitro* findings where RhoA knockdown did not influence breast cancer cell proliferation, at 30 days post-implantation we did not observe any statistically significant changes in weights or expression levels of proliferation antigen ki67 in RhoA knockdown primary tumors when compared with knockdown control (Supplementary Fig. 2). We did not observe any statistically significant changes in expression levels of cleaved caspase-3 in RhoA knockdown primary tumors as well (Supplementary Fig. 2). On the contrary, bioluminescent imaging of mice at 29 days post-implantation revealed possible distant metastases in mice implanted with the highest RhoA knockdown (shRhoA 32) 4T1 cells (Fig. 2d) showing a dose-dependent effect of RhoA knockdown on cancer metastasis. Indeed, mice implanted with shRhoA 32 knockdown 4T1 cells exhibited a significantly higher lung metastasis burden compared to other groups during histology analysis (Fig. 2e,f). In a complementary approach, we also down regulated the activity of RhoA in 4T1 cells by stably expressing the dominant negative RhoAT19N allele. Significantly, expression of RhoAT19N had the same effect on lung metastasis as the knockdown of RhoA expression in 4T1 cells and with a minimal effect on tumor weights (Supplementary Fig. 2). Collectively, these findings suggested that RhoA could act as a physiological suppressor of breast cancer lung metastasis in mice.

### RhoA suppresses breast cancer sentinel lymph node metastasis in mice

It has been thought that invasive breast cancer cells from the primary tumor gain access to the systemic circulation through intra-tumoral and peripheral blood vasculature. However, with novel and more sensitive research technologies, scientists have identified that for breast cancer, the major systemic metastasis route is through the sentinel lymph node (SLN). Once tumor cells reach the SLN, they can access the systemic circulation directly without first passing down to the next lymph node^21,22^.

**Figure 3:**
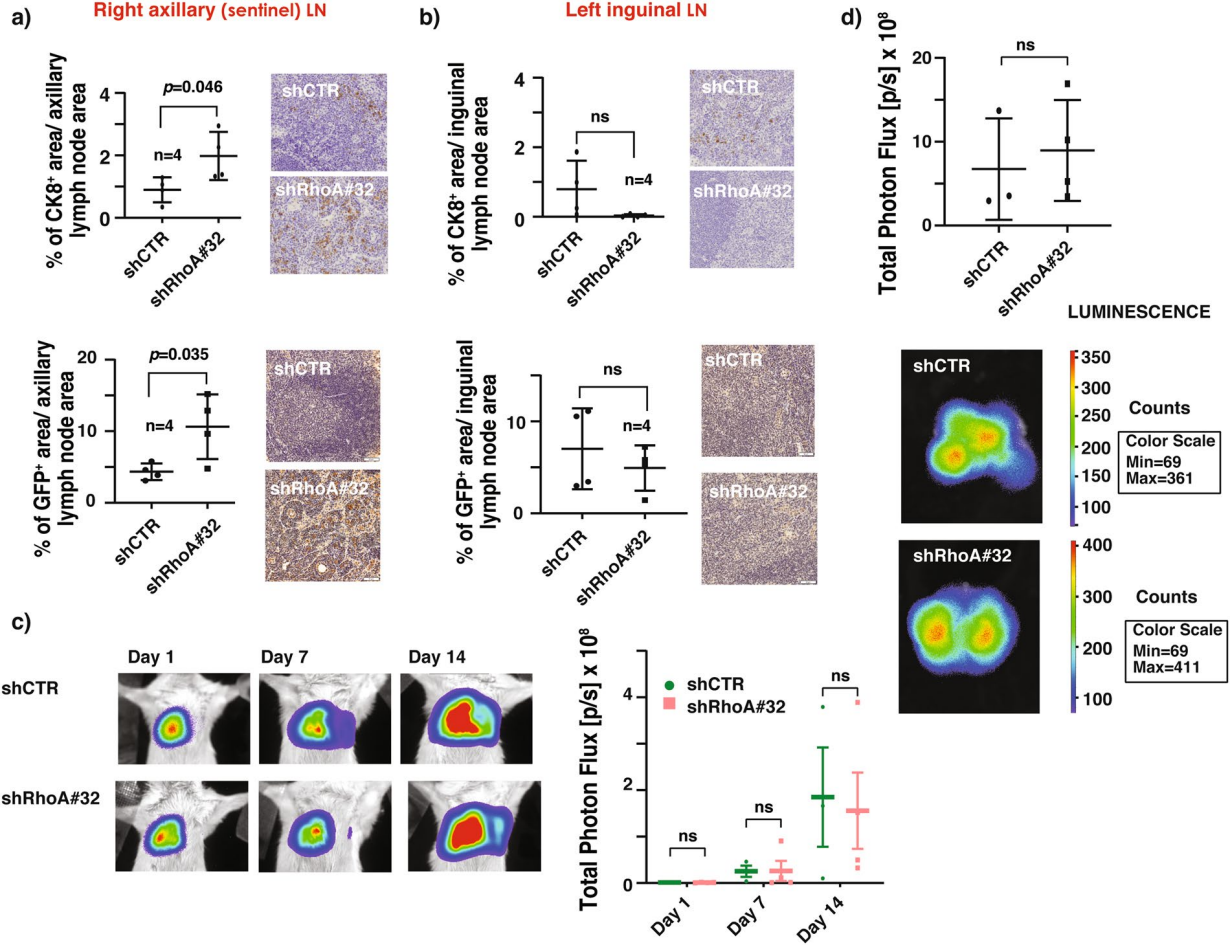
RhoA suppresses breast cancer sentinel lymph node metastasis in mice. (**a**) Representative immunohistochemical (IHC) staining images of right axillary lymph node sections of mice orthotopically injected with 4T1 GFP-LUC cells carrying indicated lentiviral modifications, stained with antibodies for CK8 (top, right) and GFP (bottom, right). Percentage of CK8^+^ area (top, left) and GFP^+^ area (bottom, left) per axillary lymph node area (mean ± SE) quantification of IHC images. N = 4. (**b**) Representative IHC staining images of left inguinal lymph node sections of above mice, stained with antibodies for CK8 (top, right) and GFP (bottom, right). Percentage of CK8^+^ area (top, left) and GFP^+^ area (bottom, left) per inguinal lymph node area (mean ± SE) quantification of IHC images. N = 4. (**c**) Representative BLI images of mice tail-vein injected with 4T1 GFP-LUC cells carrying indicated lentiviral modifications, imaged 1, 7 and 14 days after the injections showing the kinetics of lung tumor formation (left). Total photon flux quantification (mean ± SE) of BLI images shown on left (right). N = 3 for both groups. (**d**) Representative *ex vivo* BLI images of lungs harvested from above mice imaged 19 days after the tail-vein injections showing the total lung tumor burden (bottom). Total photon flux quantification (mean ± SE) of *ex vivo* lung BLI images shown below (up). N = 3, ns- not significant *P <  0.05, unpaired Student’s t-test (two-tailed).

It is possible that RhoA suppresses the lung metastasis of 4T1 breast cancer cells by interfering with the access of the cancer cells to the SLN. To examine this possibility, four weeks after implantation, we harvested the draining/sentinel lymph nodes (right axillary LN) from the cancer cells injected sites and processed them for immunohistochemical (IHC) analysis. As a control, we also harvested lymph nodes (left inguinal LN) on the contralateral side of the primary tumor. IHC analysis of the SLNs with an epithelial cell specific CK8 antibody revealed that the shRhoA 32 knockdown group mice have higher CK8 staining compared to the control mice, while no statistical significance in CK8 staining in the inguinal lymph nodes was observed between RhoA knockdown and control knockdown mice (Fig. 3a). Similar results were observed with a GFP antibody, which specifically stained the 4T1 cancer cells (Fig. 3b). Collectively, these observations suggested that when the RhoA expression is reduced, invasive 4T1 cells reached the axillary sentinel lymph node more efficiently than the control cells and readily gain access to the systemic circulation for spreading into other organs including the lungs.

Metastasis is a complex multiple-step process. In addition to invading the draining lymph node and entering the circulation, the circulating cancer cells must survive the hostile environmental conditions inside the blood vessels until extravasating into a distant tissue for metastatic colonization. To investigate whether RhoA also suppresses the later steps of the metastasis cascade, we tail-vein injected RhoA knockdown or control knockdown 4T1 GFP-LUC cells in Balb/c mice to bypass the early stages of metastasis. Mice were monitored at days 1, 7 and 14 post-injection for lung tumor burden by bioluminescent imaging. As shown in Fig. 3c,d, both the kinetics of lung tumor formation and extent of the tumor burden showed no significant difference between RhoA knockdown and control group mice suggesting that RhoA may not have a role in cancer cell extravasation and colonization.

### RhoA suppresses breast cancer cell invasion by modulating the tumor microenvironment

**Figure 4:**
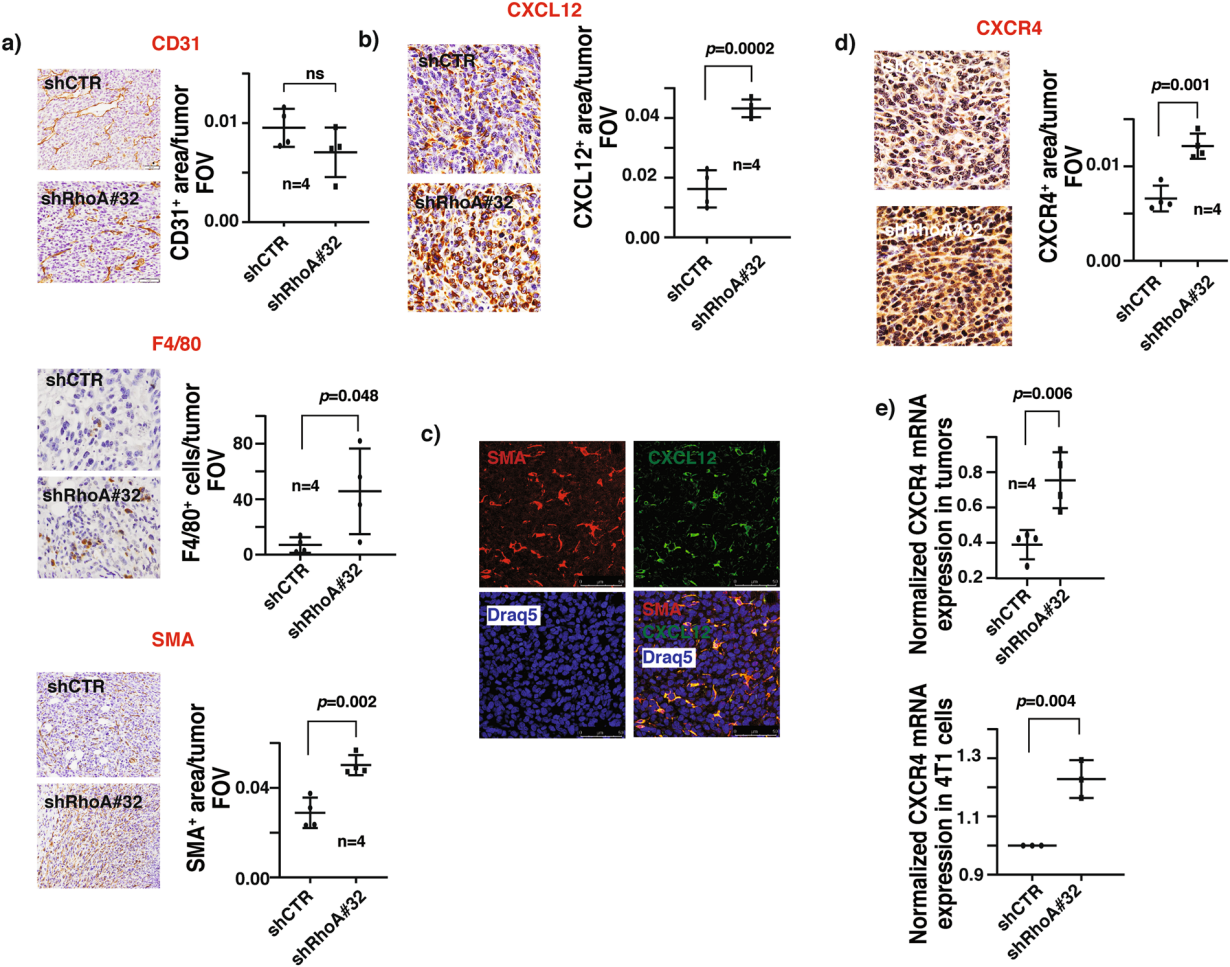
RhoA modulates the CXCR4-CXCL12 axis in breast tumors. (**a**) Representative immunohistochemical (IHC) staining images of breast primary tumor sections of mice orthotopically injected with 4T1 GFP-LUC cells carrying indicated lentiviral modifications, stained with antibodies for CD31 (top, left), F4/80 (middle, left) and α-SMA (bottom, left). CD31^+^ area (top, right), F4/80^+^ cells (middle, right) and α-SMA^+^ area (bottom, right) per tumor field of view (FOV) (mean ± SE) quantifications of IHC images. N = 4. (**b**) Representative IHC staining images of breast primary tumor sections of above mice stained with CXCL12 antibody (left). CXCL12^+^ area per tumor FOV (mean ± SE) quantification of IHC images. N = 4. (**c)** Representative immunofluorescent images of breast primary tumor sections of above mice co-stained with α-SMA, CXCL12 and DRAQ5 nuclear stain, showing the co-localization of α-SMA and CXCL12 signals. Scale bar: 50 µm. (**d**) Representative IHC staining images of breast primary tumor sections of above mice stained with CXCR4 antibody (left). CXCR4^+^ area per tumor FOV (mean ± SE) quantification of IHC images. N = 4. (**e)** Relative mCXCR4 mRNA levels normalized with mActin (mean ± SE), as quantified by qRT-PCR in primary breast tumors of above mice (top) and in 4T1 GFP-LUC cells carrying indicated lentiviral modifications (bottom). N = 4, ns- not significant *P <  0.05, unpaired Student’s t-test (two-tailed).

Constant bidirectional communication between cancer cells and tumor microenvironment (TME) plays a major impact on cancer growth and metastasis^23^. It is possible that reduced RhoA expression promotes cancer cell dissemination into sentinel lymph nodes by modulating this dynamic interplay. To investigate this hypothesis, we assessed the major TME components in the control and RhoA knockdown primary tumors harvested from the Balb/c mice 30 days after the orthotopic implantation. TME is mainly composed of activated fibroblasts called cancer-associated fibroblasts (CAF), adaptive and innate immune cells, and vascular endothelial cells. We examined the presence of vascular endothelial cells, CAFs, B-lymphocytes and macrophages in these primary tumors by IHC staining with CD31, α-SMA, CD19, and F4/80 specific antibodies, respectively. Though no differences were detected in CD31 and CD19 expression between the two groups, a significant elevation was detected in α-SMA and F4/80 expression in RhoA knockdown tumors compared to control tumors, suggesting an increased infiltration of CAFs and macrophages in RhoA knockdown tumors, respectively (Fig. 4a and Supplementary Fig. 3).

CAFs are known to drive the tumorigenesis and metastasis phenotype by secreting pro-tumorigenic chemokines, and by enhancing tumor stiffness^24^. While we did not detect any significant differences in collagen content/fibrosis between control and RhoA knockdown tumors as measured by Masson’s trichrome staining, IHC staining with pro-tumorigenic chemokine CXCL12 specific antibody revealed that RhoA knockdown tumors are significantly enriched in CXCL12 expression (Fig. 4b and Supplementary Fig. 3). To identify the source of CXCL12 in the TME, we double-stained the RhoA knockdown tumors with α-SMA and CXCL12 antibodies. We observed that the CXCL12 staining was predominantly co-localized with the staining of α-SMA suggesting that CXCL12 detected in the TME was mainly secreted by the infiltrated CAFs (Fig. 4c). In agreement with this line of thinking, we failed to detect the expression of CXCL12 in 4T1 cells by qRT-PCR (data not shown). The predominant receptor for CXCL12 is CXCR4. As expected, the expression levels of CXCR4 were also enhanced in RhoA knockdown tumors as measured by IHC staining and qRT-PCR RNA analysis (Fig. 4d,e). Apparently, the enhanced CXCR4 expression is an autonomous effect of the downregulation of RhoA expression in 4T1 cancer cells (Fig. 4e).

**Figure 5:**
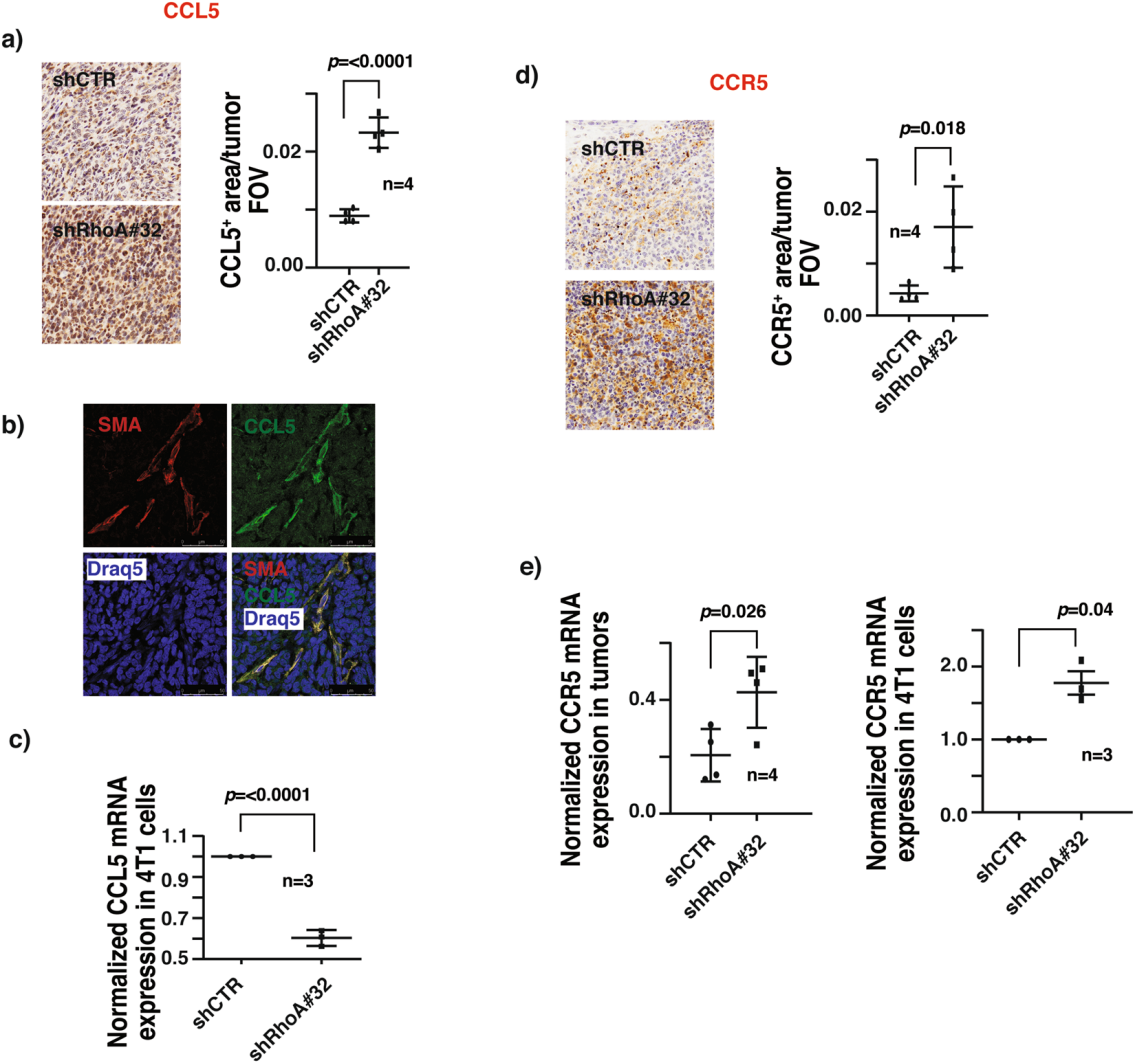
RhoA modulates the CCR5-CCL5 axis in breast tumors. (**a**) Representative IHC staining images of breast primary tumor sections of mice orthotopically injected with 4T1 GFP-LUC cells carrying indicated lentiviral modifications, stained with CCL5 antibody (left). CCL5^+^ area per tumor FOV (mean ± SE) quantification of IHC images. N = 4. (**b)** Representative immunofluorescent images of breast primary tumor sections of above mice co-stained with α-SMA, CCL5, and DRAQ5 nuclear stain, showing the co-localization of α-SMA and CCL5 signals. Scale bar:25 µm. (**c**) Relative mCCL5 mRNA levels normalized with mActin (mean ± SE), as quantified by qRT-PCR in 4T1 GFP-LUC cells carrying indicated lentiviral modifications. (**d)** Representative IHC staining images of breast primary tumor sections of above mice stained with CCR5 antibody (left). CCR5^+^ area per tumor FOV (mean ± SE) quantification of IHC images. N = 4. (**e)** Relative mCCR5 mRNA levels normalized with mActin (mean ± SE), as quantified by qRT-PCR in primary breast tumors of above mice (left) and in 4T1 GFP-LUC cells carrying indicated lentiviral modifications (right). N = 4, ns- not significant *P <  0.05, unpaired Student’s t-test (two-tailed).

Tumor-associated macrophages (TAMs) were shown to promote tumor progression, angiogenesis and lung metastasis in several breast cancer *in vivo* studies^25,26^, and associated with poor patient survival^27^. During tumor progression, secretion of tumor-derived chemoattractant chemokines leads to the recruitment of circulating monocytes into breast tumors. The infiltrated monocytes will subsequently differentiate into tumor associated-macrophages^28^. It is possible that the increased number of TAMs is a consequence of increased expression of CCL5 in RhoA knockdown tumors. Truly, RhoA knockdown tumors were significantly enriched in CCL5 expression (Fig. 5a). In addition to secreting CXCL12, CAFs have also been shown to be a key source of CCL5 in tumors^29^. Indeed, CAFs but not the cancer cells, are the major contributors of CCL5 in RhoA knockdown tumors as assessed by immunofluorescent staining and qRT-PCR (Fig. 5b,c).

Paracrine activities of intra-tumoral CCL5 through its cognate receptor CCR5 are reported to promote breast tumor progression, and LN and thoracic metastasis^30–32^. It is possible that the observed increase in LN and lung metastases in RhoA knockdown mice is caused by activated CCL5-CCR5 signaling in primary tumors. In agreement with this line of reasoning, we observed the expression levels of CCR5 were increased in RhoA knockdown tumors and the increased CCR5 expression levels are partly due to an increase in CCR5 transcripts in cancer cells (Fig. 5d,e).

## Discussion

In this study, we investigated the role of RhoA in breast cancer cell proliferation, invasion, and metastasis. Stable knockdown of RhoA expression had no effect on breast cancer cell proliferation but increased *in vitro* cell invasion and metastasis. The reported effects of RhoA shRNA-mediated knockdown on breast cancer cell invasion *in vitro* are mixed. While we and others observed that RhoA has an anti-invasive role^14,15,33^, published reports indicating that RhoA has a pro-invasive role in breast cancer exist^34–36^. At present, the mechanism of how RhoA can have opposite effects on breast cancer cell invasion *in vitro* is not known. RhoA GTPase has several downstream effectors and some of them reported to have opposing effects on cancer cells invasion^37^. Therefore, it is plausible that, depending on the experimental conditions, knocking down the expression of RhoA can have preferred effects on certain downstream RhoA effectors. However, despite the described opposite effects of RhoA on *in vitro* breast cancer cells invasion, the effects of specific RhoA shRNA knockdown on breast cancer metastasis has never been formally addressed with an orthotopic transplantation immune-competent mouse model until now. The results of our *in vitro* invasion assays align rightly with the *in vivo* mouse experiments strongly suggesting the causal role of RhoA as a breast cancer metastasis suppressor. Among the various effectors, mDia and ROCK are two best-described effectors that are important for RhoA-mediated regulation of cancer cell invasion/metastasis by stabilizing adherens junctions, regulating myosin light chain 2 (MLC2) phosphorylation and focal adhesion dynamics^38–42^. It remains to be determined if RhoA inhibits breast cancer cell metastasis by targeting adherens junctions, MLC2 phosphorylation, and/or focal adhesions.

In addition to cancer cell-autonomous functions, continuous heterotypic communications between cancer cells, ECM, and their surrounding non-malignant cells are also vital for disease progression and metastatic dissemination^23^. Fibroblasts are the most common non-malignant cells in the tumor. In normal tissues, they usually exist in a resting/quiescent state and are activated in response to a plethora of signaling cues. Activated fibroblasts are highly proliferative and metabolically active and identified by their expression of markers like α-SMA and fibroblast activation protein (FAP). Activated fibroblasts in tumor stroma are termed cancer-associated fibroblasts (CAFs), which are generally pro-tumorigenic with a dynamic and complex secretory phenotype including ECM molecules (tenascin C, collagen), growth factors (VEGFA, PDGF, HGF), cytokines, and chemokines (TNF, IFNγ, CXCL12, CCL5, IL-6, IL-8)^24^. Using the 4T1 triple negative breast cancer mouse model, we observed an inverse correlation between RhoA expression and infiltration of α-SMA positive CAFs. Consistent with increased infiltrated CAFs, RhoA knockdown tumors were also highly enriched with CAFs-secreted CXCL12. The reduced RhoA activity resulted in increased expression of the cognate receptor, CXCR4, for CXCL12 in 4T1 cancer cells.

CAFs-secreted CXCL12 drives tumor progression and metastasis through two possible mechanisms. It mediates the chemotactic recruitment of endothelial progenitor cells (EPC) to promote tumor angiogenesis. It may also enhance the invasive capacity of CXCR4-expressing breast cancer cells through paracrine stimulation^43^. Since RhoA knockdown had no observable effect on tumor angiogenesis, it is possible that the increased CXCL12 expression might drive the paracrine stimulation of the RhoA knockdown cancer cells’ invasiveness. Therefore, our study suggests that RhoA suppresses breast cancer cell metastatic dissemination by suppressing the invasiveness of tumor cells through reduced expression of the tumor CXCR4 receptor with a subsequent reduction of CAFs in the TME.

Similarly, RhoA knockdown primary tumors displayed a significant F4/80 positive macrophage infiltration. This was correlated with an increased expression of stromal-derived CCL5, a well-established chemokine important in recruiting and converting CCR5 positive monocytes into TAMs. In addition to CCR5, monocytes also express CCR2, which is the main receptor for another important immune cell recruiting chemokine CCL2^44^. The expression of CCR5, the cognate receptor for CCL5 was likewise found to be upregulated in RhoA knockdown 4T1 cancer cells. However, the effects of RhoA knockdown on CCR2 expression is not as significant as the effects on CCR5 (unpublished results). It is therefore possible that the upregulation of CCR5 but not CCR2 expression is the major cause of increased F4/80 positive macrophage infiltration in RhoA knockdown tumors. In addition to CXCL12, CAFs were also a source of increased CCL5 expression in RhoA knockdown tumors. It is therefore likely that the increased co-expression of CXCR4 and CCR5 in RhoA knockdown cancer cells facilitates the recruitment of CXCL12/CCL5 secreting CAFs and later the TAMs into the TME. Like CAFs, TAMs have been shown to be pro-tumorigenic and pro-metastatic. Since RhoA knockdown tumors with significant CAFs and TAMs infiltration showed relatively increased lymph node and lung metastases formation, it is therefore possible that reduced RhoA activity in tumor cells promotes its metastatic dissemination by activating both the CXCL12-CXCR4 and CCL5-CCR5 signaling pathways.

Metastatic dissemination and distant colonization are inefficient processes with multiple sequential steps. The systemic metastasis of breast cancer was long thought to be through the intratumoral and peritumoral blood vasculature, even though lymph node metastasis was often associated with tumor aggressiveness and poorer prognosis^45^. It was long believed that LN metastases enter systemic circulation by traveling from SLN to the next through efferent lymphatics until they drain into the thoracic duct and through the neck lympho-vascular anastomosis into the internal jugular vein^45^. However, recent studies using intravital imaging, photo-switchable protein, and cancer cell micro-infusion showed that cancer cells in the SLN are a significant source for distant lung metastases and lymph node-residing tumor cells disseminate by efficiently invading LN blood vasculature rather than by transiting through efferent lymphatic vessels^21,22^. In the present study, we detected more cancer cells in the SLNs but not in the contralateral lymph nodes of RhoA knockdown mice compared to the control group. Cancer cells tail-vein injection experiments designed to bypass the early steps revealed no difference in cancer cells extravasation and lung colonization between RhoA knockdown and control groups. Taken together, our study suggest that RhoA suppresses the cancer cells’ initial steps of local invasion into SLNs, and subsequent intravasation into LN blood vasculature while has no observable role in the later steps of the metastasis cascade.

Knockdown of RhoA expression increases CCR5 and CXCR4 transcripts in 4T1 cancer cells suggesting a possible transcription-regulatory function of RhoA. RhoA GTPases are known for its functions in cytoskeleton dynamics, regulating cell polarity and cell migration and cell cycle progression^1^. However, a lesser-known function of RhoA is its ability to regulate several transcriptional signaling pathways such as NFκB signaling^46^, GATA-4 signaling^47^, CREB signaling^48^, STAT5a signaling^49^ and AP-1 signaling^50^. Rho regulates the transcriptional activity of NFκB by a mechanism involving phosphorylation of IκBα^51^, and in breast and prostate cancer cells, NFκB directly upregulates CXCR4 mRNA expression and stability^52,53^. Although NF-κB has a stimulatory role in regulating chemokines expression in general, it is still conceivable that RhoA negatively regulates NF-κB by decreasing the expression of coactivators required for optimal activity of NF-κB^54^. Similarly, it was also reported that Rho GTPases could modulate the activity of CREB, and CCR5 is a well-established transcriptional target of the cAMP/PKA/CREB pathway^48,55^. Therefore, it is possible that RhoA suppresses CCR5 and CXCR4 expression by impinging on one or more of these transcriptional signaling pathways. In a recent study, Georgouli *et al*. reported the pivotal role of RhoA-ROCK signaling in driving fast rounded-amoeboid migration in cancer cells during metastasis in a melanoma mouse model. Mechanistically upregulation of the RhoA-ROCK pathway in melanoma cancer cells stimulates the transcription and secretion of an entire cytokine transcriptional program that controls immune cell recruitment into the tumor environment^42^. At present, it is not clear whether the pro-metastatic effect of RhoA is RhoA-effector or cancer type specific.

In summary, using a triple negative breast cancer mouse model, we demonstrated that reduced RhoA expression increases breast cancer lymph node and lung metastasis. Potential tumor and metastasis suppressive ability of RhoA in breast cancer have been suggested before^14,15^. But the current study demonstrated that this is achieved by promoting a pro-tumor microenvironment, with increased CAF and macrophage infiltration, and by modulating the CCL5-CCR5 and CXCL12-CXCR4 chemokine axes at the primary tumor. To our knowledge, this is the first such mechanistic study in breast cancer, shows the ability of RhoA to suppresses chemokine receptor expression in breast tumor cells, in turn suppressing the breast cancer LN and lung metastasis.

## Materials and Methods

### Cell culture

BT20 and MDA-MB231 human breast cancer cell lines were cultured in Dulbecco’s modified Eagle’s medium (DMEM, HyClone, UT, USA) with 10% FBS (Atlanta Biologicals, GA, USA) and 1% penicillin-streptomycin (HyClone), and the 4T1 mouse breast cancer cell line was cultured in DMEM with 5% FBS, 5% calf serum (Lonza, Switzerland) and 1% penicillin-streptomycin. Cells were grown in a humidified tissue culture incubator at 37 °C in 5% CO_2_. 4T1 cells were kindly provided by Dr. Fred Miller (Karmanos Cancer Institute, MI), and MDA-MB231 cells and BT20 cells were purchased from ATCC.

### Clonogenic growth assay

BT20 (1500 cells/well) and MDA-MB231 (2500 cells/well) cells were plated on a 6 well plate in triplicates and grown for 10 days before staining. For PD0325901 (CAS# 391210-10-9) treatment, BT20 and MDA-MB231 cells were plated with 2 µM and 10 µM of PD0325901 respectively, for 10 days before staining. Cells were fixed with a 4% formaldehyde solution and stained in a 0.5% crystal violet for 24 hours before being washed with 1x PBS and imaged. The analysis was done with ImageJ software (NIH).

### Immunofluorescence with Ki67

Cells were plated on laminin-coated (Sigma, MO, USA) glass coverslips and grown until the desired confluency. Next, they were fixed with 100% methanol for 10 min, and incubated with anti-Ki67 antibody (SC-15402, Santa Cruz, CA, USA), Alexa Fluor® 546 secondary antibody (1:5000, ThermoFisher, MA, USA), and DRAQ5® (1:2000, Cell Signaling, MA, USA) nuclear stain. Fluorescence images were captured with a Leica TCS SP5 multiphoton laser scanning confocal microscope and analyzed with ImageJ software.

### Immunohistochemistry

FFPE sections were deparaffinized and rehydrated with an ethanol gradient. Antigen retrieval was performed with citrate buffer (pH 6) and tissues were blocked with 10% normal goat serum (Vector Laboratories, CA, USA) for 2 hours. Overnight primary antibody incubations were carried out with Ki67 (SC-15402, Santa Cruz, CA, USA), CD-19 (bs-4755R, Bioss, MA, USA), CK8 (TROMA-I, deposited to DSHB by Brulet, P./ Kemler, R.), E-cadherin (3195, Cell Signaling, MA, USA), GFP (ab290, Abcam, MA, USA), CD31 (ab28364, Abcam), F4/80 (ThermoFisher, MA, USA), α-SMA (19245, Cell Signaling), CCR5 (ab65850, Abcam), CXCR4 (MAB21651, R&D systems, MN, USA), CCL5 (AP-20618, Acris, MD, USA) and CXCL12 (MAB350, R&D systems). Sections were incubated with biotinylated secondary antibodies (Vector Laboratories) and ABC reagent (Vector Laboratories) and developed with DAB reagent (Sigma). Finally, they were counterstained with hematoxylin (Fisher Scientific, NH, USA), dehydrated with an ethanol gradient, and mounted with Permount™ (Fisher Scientific). Whole slides were scanned with an Olympus slide scanner. To quantify the IHC positive signal, ImageJ software was used. Briefly, ten random fields of view (FOV) were captured from each tumor section while avoiding necrotic and non-tumor areas. Positive signal area or positive number of cells were measured using the ImageJ for each FOV. The average positive signal area or positive number of cells were reported as positive area or positive number of cell per tumor FOV. For co-localization studies, after primary antibody incubation, sections were incubated with corresponding Alexa Fluor® secondary antibodies (1:2000, 1 hour, ThermoFisher) and DRAQ5® (1:2000, 10 minutes, Cell Signaling, MA, USA) nuclear stain. Fluorescence images were captured with a Leica TCS SP5 multiphoton laser scanning confocal microscope.

### *In vitro* Matrigel Invasion assay

The PET membranes (8 µm pore size) of FluoroBlok™ cell culture inserts (351152, Corning, NY, USA) were coated with 90 µL of diluted Matrigel (0.3 mg/ml) (356234, Corning), and incubated at 37 °C for 2–3 hours until solidified. Next, 1 × 10^4^ of 4T1, BT20 or MDA-MB231 cells suspended in serum-free DMEM media were seeded on the solidified Matrigel layer. Then, 700 µL of chemo-attractant medium (DMEM, 1% P/S and 10% FBS) was added to the lower chambers (353504, multiwell 24 well companion plate, Corning), and the plate was incubated in a 37 °C incubator. After 24 hours of incubation, the insert bottoms were dipped in 1X PBS and stained in diluted Calcein AM (354217, Corning) in PBS for 10 min. Fluorescence images of invaded cells were captured with an EVOS inverted microscope, and analysis was done with ImageJ software.

### Mammary fat pad injection for spontaneous metastasis assay

The detailed procedure for mammary fat pad injection was reported before^56^. For all our studies, BALB/c female mice (Taconic, NY, USA) of 5 weeks old were used and 100 000 of 4T1 GFP-LUC control knockdown and RhoA knockdown cells were injected. After 29 days of injections, whole animals were imaged for luciferase bioluminescence (BLI) with a Xenogen IVIS system as follows: Fresh D-luciferin (LUCK-1G, GoldBio, MO, USA) working solution of 15 mg/ml was prepared in 1x PBS, and it was filter sterilized. Mice were inoculated with a 10 µl of luciferin solution per gram of body weight as an IP injection. After 10 min, they were anesthetized with Isoflurane and imaged. Images were processed and quantified with a Living Image 3.2 software.

After 30 days of injections, mice were euthanized, and blood and tissues were collected and processed for RNA or FFPE histology analysis. Macro-metastases on the lung surface were imaged and counted.

The Department of Laboratory Animal Resources at the University of Toledo Health Science Campus is accredited by the Association for the Assessment and Accreditation of Laboratory Animal Care International (AAALAC) and operates in full compliance with the OLAW/PHS policy on the Humane Care and Use of Laboratory Animals and the USDA Animal Welfare Act. All animal protocols used in this study were approved by the University of Toledo Institutional Animal Care and Use Committee (IACUC) and all experiments were performed in accordance with relevant guidelines and regulations in the approved protocols.

### Experimental metastasis assay

Tail vein (IV) injections for experimental metastasis were performed as reported before^56^. For our studies, 200 000 of 4T1 GFP-LUC control knockdown and RhoA knockdown cells were tail vein injected. Whole animals were imaged on days 1, 7 and 14 for BLI as described above. After 19 days of injections, mice were euthanized, and lungs were harvested and *ex vivo* BLI were performed as follows: Fresh D-luciferin working solution of 300 µg/ml was prepared in 1x PBS. Lungs were soaked in 1 ml of luciferin solution for 5 min in a 24 well plate and imaged with a Xenogen IVIS system. Images were processed and quantified with a Living Image 3.2 software.

### Real-time PCR analysis

Total RNA samples were prepared from cultured cells and mice tumor tissue samples with Qiagen™ RNA preparation kits. These RNAs were used to synthesize cDNA (M-MLV kit, Invitrogen, MA, USA). Real-time PCR reactions were performed with SyBR green reagent (Qiagen, MD, USA) and appropriate RT-PCR primers. Primer details are as follows; mCXCR4 F-GACTGGCATAGTCGGCAATG, R-AGAAGGGGAGTGTGATGACAAA; mCCR5 F-TTTTCAAGGGTCAGTTCCGAC, R-GGAAGACCATCATGTTACCCAC; mCXCL12 F-TGCATCAGTGACGGTAAACCA, R-TTCTTCAGCCGTGCAACAATC; mCCL5 F-GCTGCTTTGCCTACCTCTCC, R-TCGAGTGACAAACACGACTGC; mCYCLOPHILLINA F-GAGCTGTTTGCAGACAAAGTTC, R-CCCTGGCACATGAATCCTGG; mβ-ACTIN F-ATCTGGCACCAGACCTTCTACAATGAGCTGCG, R-CGTCATACTCCTGCTTGCTGATCCACATCTGC.

### Western blotting

Total cell lysates were prepared with the lysis buffer (20 mM Tris, pH 7.4, 150 mM NaCl, 2 mM EDTA, and 1% Triton X-100), and proteins were separated with sodium dodecyl sulfate-polyacrylamide gel electrophoresis (SDS-PAGE). Then, separated proteins were electrophoretically transferred from the gel to a polyvinylidene fluoride membrane (Millipore, MA, USA). Membranes were blocked and incubated overnight with primary antibodies diluted in PBS (pH 7.4), 0.2% Tween-20, 5% bovine serum albumin and 0.002% sodium azide. Primary antibodies used for western analyses were RhoA (1:1000, Cell Signaling) and tubulin (1:2000, Sigma). Then, they were incubated with the appropriate horseradish peroxidase-conjugated secondary antibodies (1:2000, Cell Signaling) and developed with Immobilon western chemiluminescence substrate (Millipore) in a Bio-Rad ChemiDoc EQ system.

### Statistical analysis

Statistical calculations with two-tailed Student’s t-test were done using GraphPad Prism software. All the data are presented as means with error bars representing standard error.

## Supplementary information

Supplementary figures

## Data Availability

No datasets were generated or analyzed during the current study.
