# Supplementary material for: Reduced RhoA expression enhances breast cancer metastasis with a concomitant increase in CCR5 and CXCR4 chemokines signaling
Source: Sci Rep. 2019 Nov 8;9:16351. doi: 10.1038/s41598-019-52746-w (PMC6841971; doi:10.1038/s41598-019-52746-w)
Supplement: Supplementary file 1 — Supplementary figures [file 41598_2019_52746_MOESM1_ESM.docx]

**Reduced RhoA expression enhances breast cancer metastasis with a concomitant increase in CCR5 and CXCR4 chemokines signaling**

Gardiyawasam Kalpana^1^, Christopher Figy^1^, Miranda Yeung^1^, Kam C. Yeung^1*^


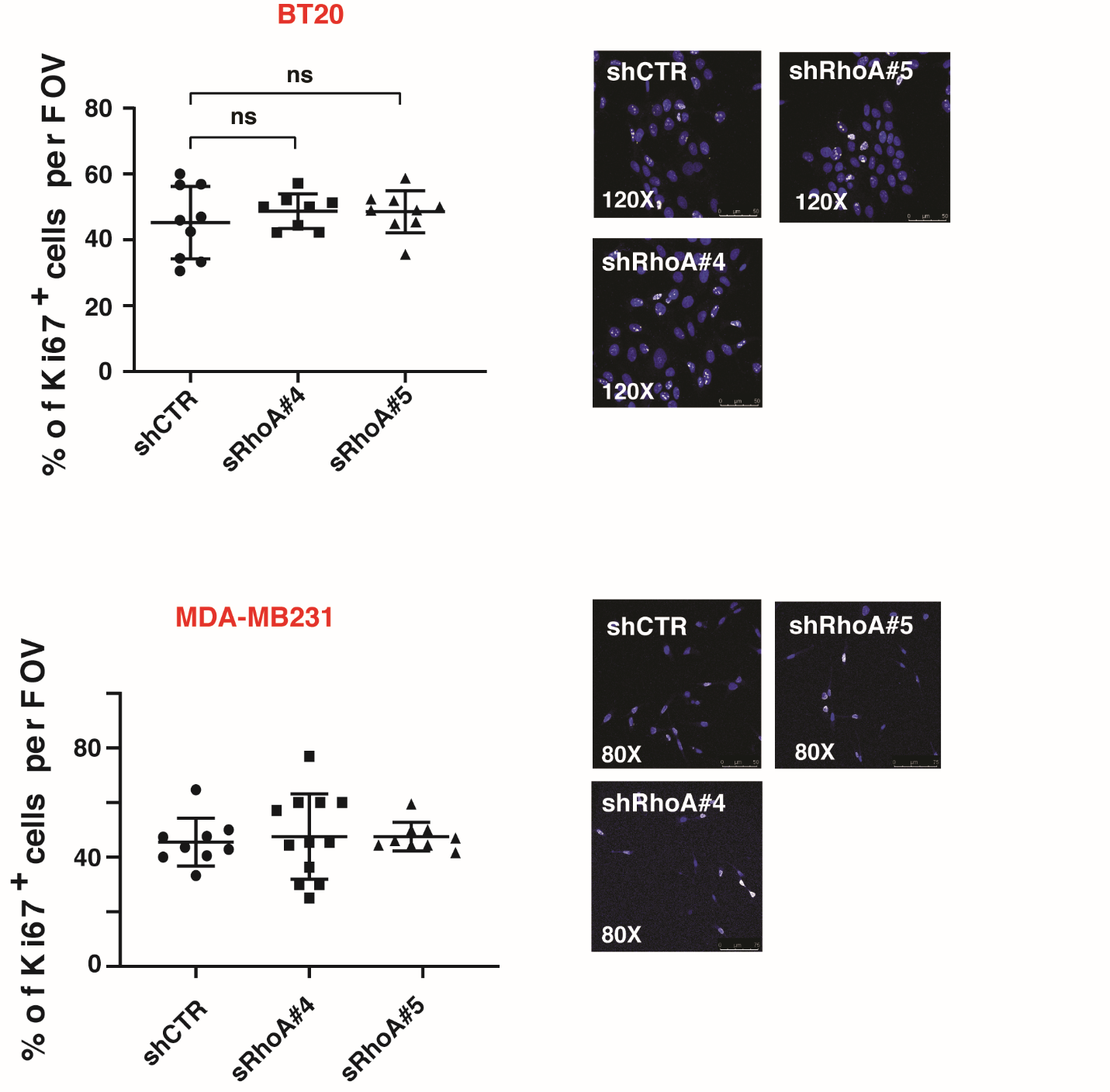


**Supplementary Figure 1.** Effect of RhoA on breast cancer cell proliferation. Representative immunofluorescent images of BT20 (top, right) and MDA-MB231 (bottom, right) cells co-stained with Ki67 and DRAQ5 nuclear stain, showing the co-localization of Ki67 and nuclei. Percentage of Ki67^+^ cells per field of view (FOV) (mean $\pm$ SE) quantification of immunofluorescent images of BT20 (top, left) and MDA-MB231 (bottom, left). ns- not significant *P$<$0.05, unpaired Student's t-test (two-tailed).


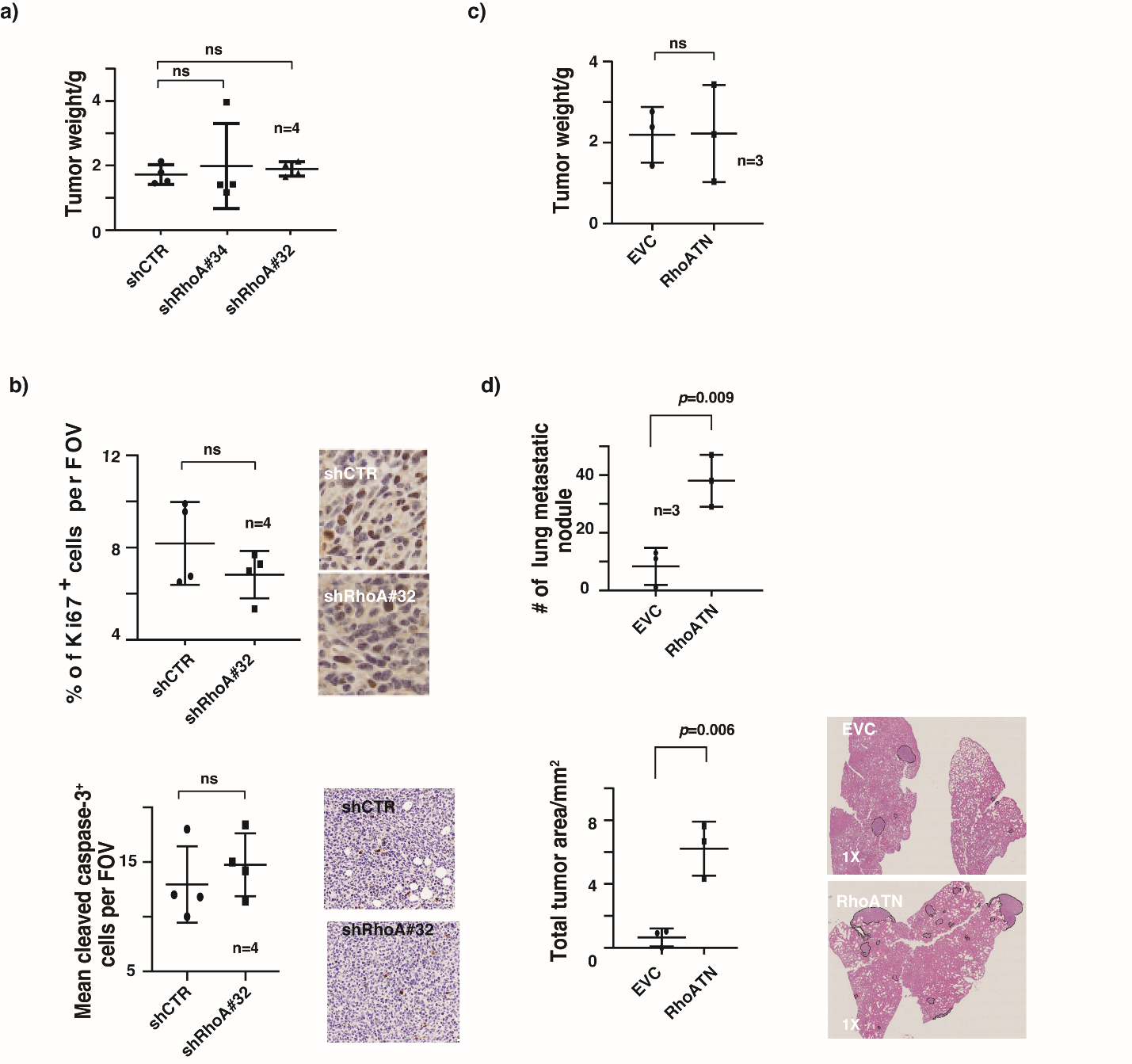


**Supplementary Figure 2.** Effect of RhoA on breast tumor formation and metastasis *in vivo*. **a** Weight of the breast primary tumors (mean $\pm$ SE) of mice orthotopically injected with 4T1 GFP-LUC cells carrying indicated lentiviral modifications, in grams. N=4. **b** Representative immunohistochemical (IHC) staining images of breast primary tumor sections of above mice, stained with Ki67 antibody (bottom). Percentage of Ki67^+^ cells per field of view (FOV) (mean $\pm$ SE) quantification of IHC images (top). N=4. **c** Weight of the breast primary tumors (mean $\pm$ SE) of mice orthotopically injected with 4T1 GFP-LUC cells carrying indicated lentiviral modifications, in grams. N=4. **d** Number of lung metastatic nodules (mean $\pm$ SE) of mice orthotopically injected with 4T1 GFP-LUC cells carrying indicated lentiviral modifications. N=3 (top). Representative hematoxylin and eosin (H&E) staining of lung cross sections from above lungs showing metastases highlighted with black (bottom, right) and total metastases tumor area (mean $\pm$ SE) quantification of the H&E images (bottom, left). N=3, ns- not significant *P$<$0.05, unpaired Student's t-test (two-tailed).


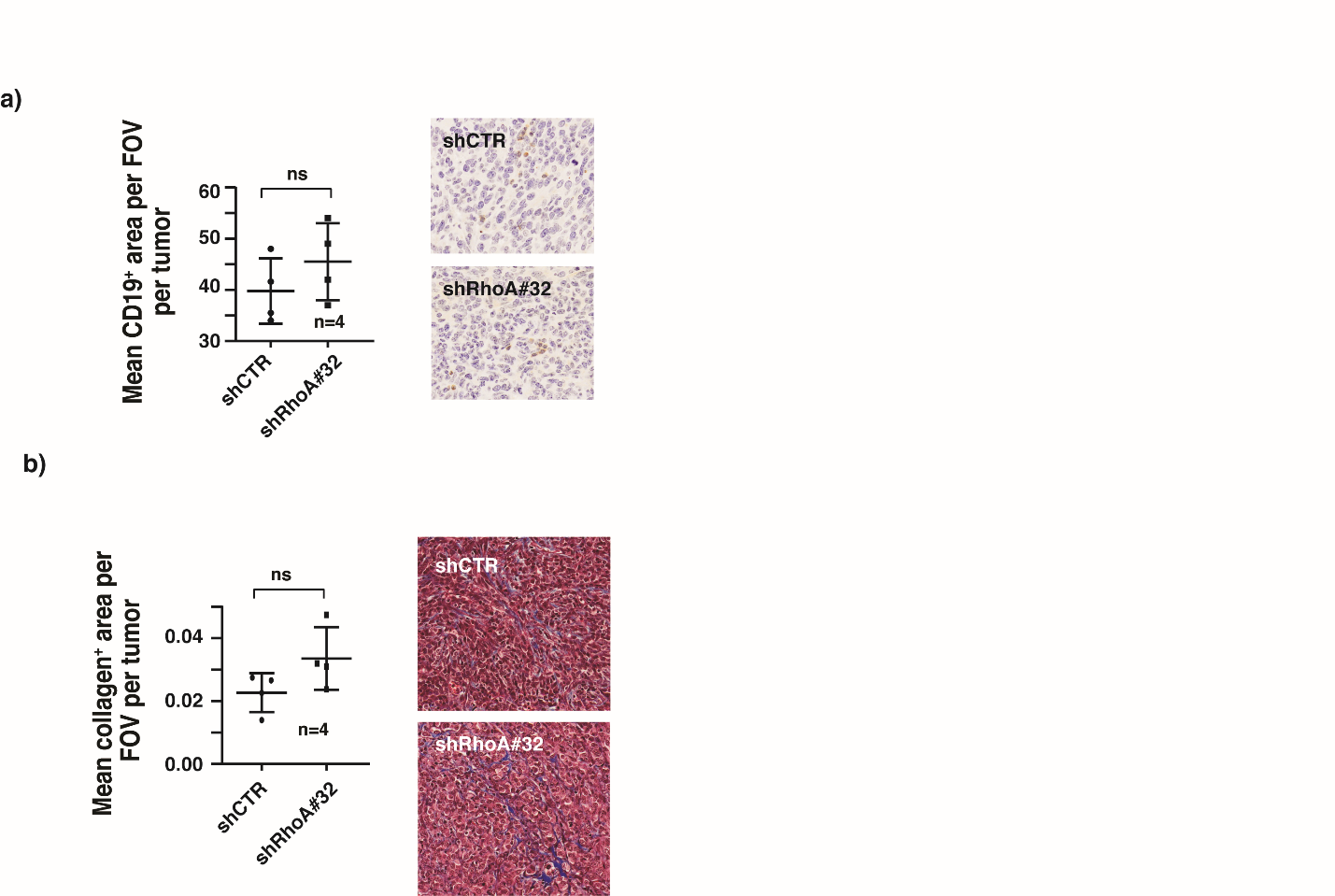


**Supplementary Figure 3.** Effect of RhoA on primary breast tumor characteristics. **a** Representative immunohistochemical (IHC) staining images of breast primary tumor sections of mice orthotopically injected with 4T1 GFP-LUC cells carrying indicated lentiviral modifications, stained with CD19 (right). CD19^+^ area per tumor field of view (FOV) (mean $\pm$ SE) quantification of IHC images. N=4. **b** Representative trichrome staining images of breast primary tumor sections of above mice (right). Collagen^+^ area per tumor field of view (FOV) (mean $\pm$ SE) quantification of trichrome images. N=4, ns- not significant *P$<$0.05, unpaired Student's t-test (two-tailed).
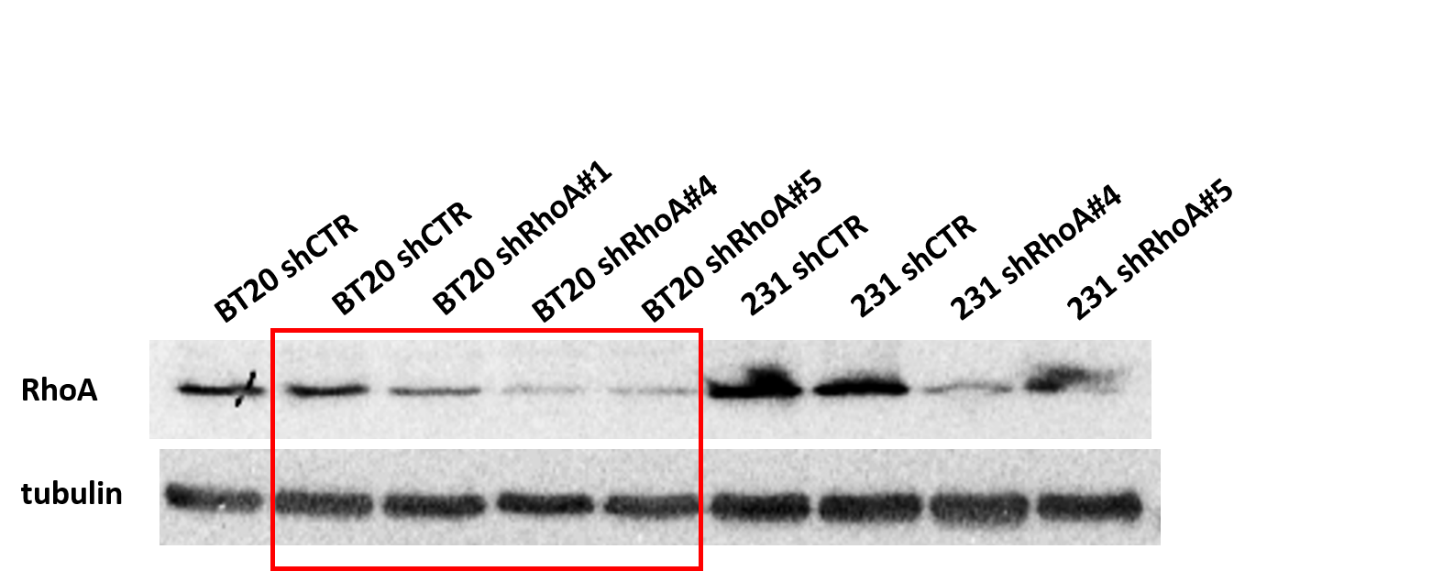


**Supplementary Figure 4.** Level of RhoA knockdown in BT20 cells. The complete blots from the western analysis of BT20 and MDA-MB231 cells carrying indicated lentiviral modifications, immunoblotted with RhoA and Tubulin specific antibodies. The red square is showing the cropped region of the blots to include in the figure 1a of the original article.


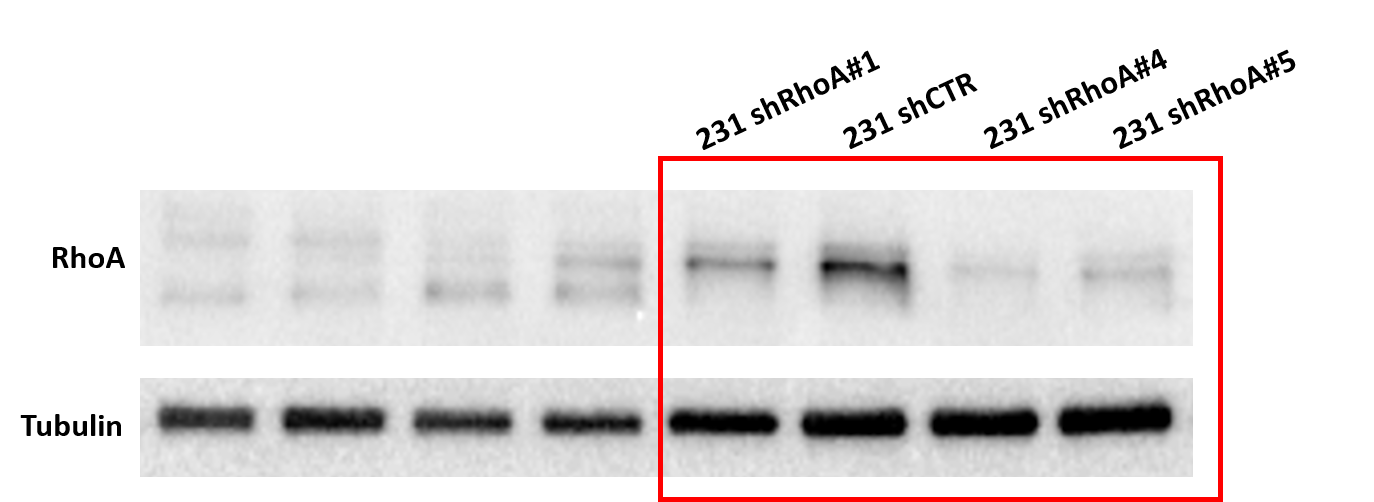


**Supplementary Figure 5.** Level of RhoA knockdown in MDA-MB231 cells. The complete blots from the western analysis of MDA-MB231 cells carrying indicated lentiviral modifications, immunoblotted with RhoA and Tubulin specific antibodies. The red square is showing the cropped region of the blots to include in the figure 1a of the original article.


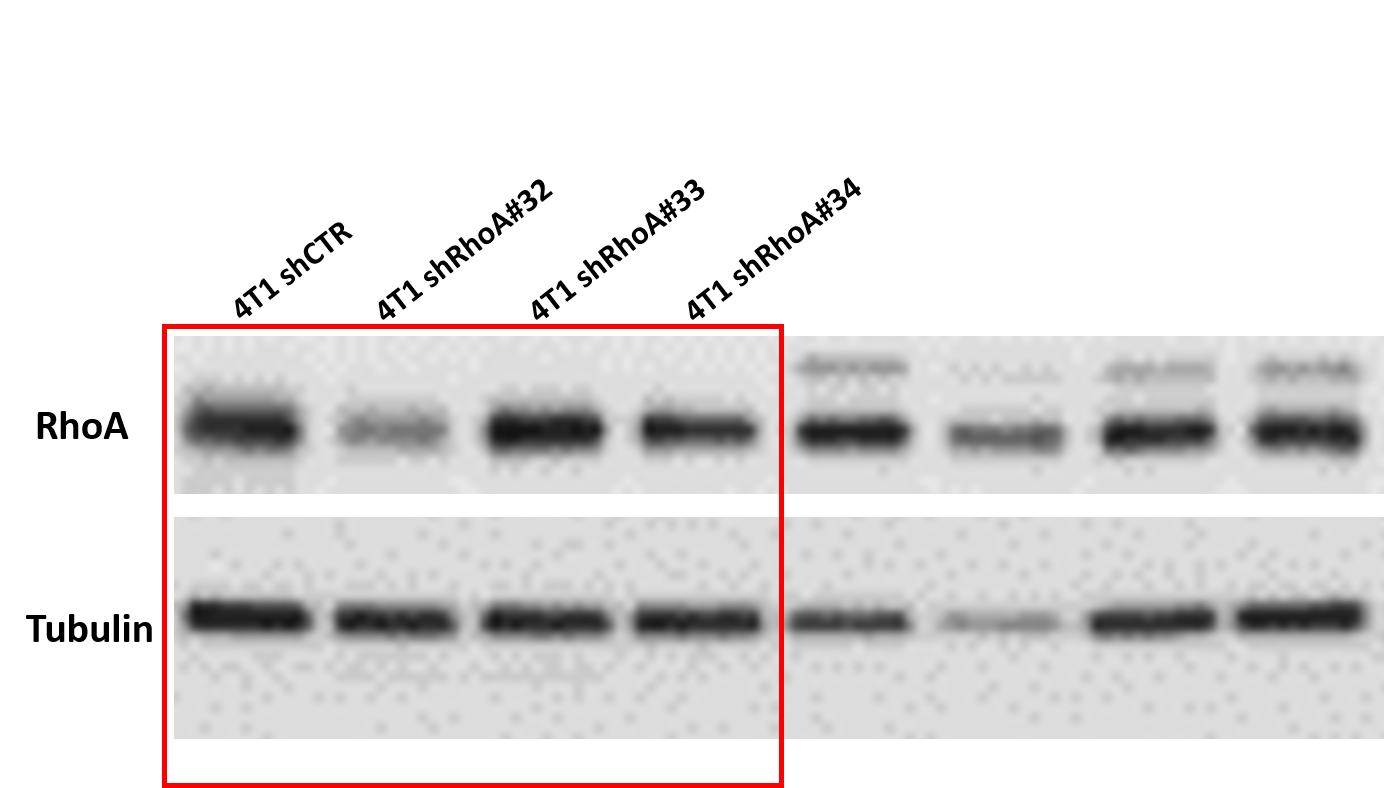


**Supplementary Figure 6.** Level of RhoA knockdown in 4T1 cells. The complete blots from the western analysis of 4T1 cells carrying indicated lentiviral modifications, immunoblotted with RhoA and Tubulin specific antibodies. The red square is showing the cropped region of the blots to include in the figure 2a of the original article.
